# ‘Do I actually even need all these tablets?’ A qualitative study exploring deprescribing decision-making for people in receipt of palliative care and their family members

**DOI:** 10.1177/02692163251327900

**Published:** 2025-04-01

**Authors:** Anna Robinson-Barella, Charlotte Lucy Richardson, Zana Bayley, Andy Husband, Rona Bojke, Andy Bojke, Rachel Quibell, Lisa Baker, Emma McDougall, Catherine Exley, Barbara Hanratty, Joanna Elverson, Jesse Jansen, Adam Todd

**Affiliations:** 1 School of Pharmacy, Newcastle University, Newcastle upon Tyne, UK; 2 Newcastle Patient Safety Research Collaborative PSRC, Newcastle University, Newcastle upon Tyne, UK; 3 Population Health Sciences Institute, Newcastle University, Newcastle upon Tyne, UK; 4 Patient and Public Involvement, Newcastle University, Newcastle upon Tyne, UK; 5 The Newcastle Hospitals Foundation Trust, Newcastle upon Tyne, UK; 6 St. Benedict’s Hospice, St. Benedict’s way, Ryhope, Sunderland, UK; 7 Northumbria Healthcare NHS Foundation Trust, Newcastle upon Tyne, UK; 8 St. Oswald’s Hospice, Regent Avenue, Newcastle upon Tyne, UK; 9 Faculty of Health, Medicine and Life Sciences, Maastricht University, Maastricht, The Netherlands

**Keywords:** Deprescriptions, palliative care, medication review, qualitative research

## Abstract

**Background:**

For people in receipt of palliative care, where polypharmacy is common and medication burden is high, there remains limited knowledge around the decision-making processes that underpin deprescribing; for example, recent deprescribing studies have focused on wider issues of identifying polypharmacy in palliative care contexts. However, little is known about the specific challenges of, and preferences towards, decision-making to support the deprescribing for people in receipt of palliative care.

**Aim:**

To explore decision-making processes that underpin deprescribing approaches, based on the experiences of people in receipt of palliative care, and their family member(s).

**Design:**

An explorative qualitative study involving in-person semi-structured interviews, analysed using reflexive thematic analysis.

**Setting/participants:**

Twenty-five semi-structured interviews were conducted with people in receipt of palliative care (*n* = 25), where 12 of these interviews were undertaken as dyads, with both the patient and a family member together. Interviews were undertaken across a range of settings, spanning: hospice outpatient day units (*n* = 11), hospice inpatient wards (*n* = 4), care home (*n* = 1) and patients’ own homes (*n* = 9), and involved people with diverse diagnoses (including: cancer 52%, heart failure 20%, motor neurone disease 12%, pulmonary fibrosis 4% and chronic obstructive pulmonary disease 4%).

**Results:**

Two overarching themes were developed – the first reflected the need to address patient understanding by ‘laying the foundations of deprescribing decision-making’. The second theme, ‘having a voice in deprescribing decision-making’, reflected desires to (pro)-actively involve patients and their family member(s) within these processes.

**Conclusion:**

There is a need to take a balanced, person-centred and shared approach to deprescribing decision-making for people receiving palliative care. Co-design strategies offer one approach to further explore this.

**What is already known on this topic?**

- Much research exists across broad contexts of deprescribing for vulnerable cohorts, with the voices of those with lived experiences at the centre.
- The deprescribing of medication aims to improve quality of life, whilst reducing tablet burden and aligning to an individual’s goals and priorities for treatment.
- Deprescribing in the context of palliative care settings is beginning to receive attention from researchers and has been regarded as a significant step towards individualised medicines management to optimise quality of life.

**What this paper adds?**

- By focusing specifically on the context of deprescribing medications for people in receipt of palliative care, this paper is the first to consider the decision-making steps that underpin this process.
- This study centres the voices of those receiving palliative care, alongside those of their family and/or caregivers, to offer perspectives from a lived-experience.

**Implications for practice, theory or policy**

- It is clear that people in receipt of palliative care, and their family/carer(s), wish to play an active role in deprescribing decision-making; consideration now needs to focus on the best approach(es) to achieve this in clinical practice.
- Continuity and familiarity with the healthcare professional team can facilitate people in receipt of palliative care to find their voice and contribute to decisions about deprescribing.
- Co-design methodologies could further inform how best to frame and approach conversations between patients, their family members/carer(s) and healthcare professionals to enable informed and shared deprescribing decision-making.

## Background

Polypharmacy, the concomitant use of multiple medications, is becoming increasingly common.^1,2^ Deprescribing has been defined as the process of tackling polypharmacy; where medications that may no longer be beneficial, or are potentially causing harm, are reduced or stopped.^3–5^ Deprescribing processes take place under the planning and supervision of healthcare professionals with the aim of improving quality of life by reducing unnecessary treatment burden and adverse events, and aligning care with the individual’s goals, values and priorities.^6–8^

Globally, the need for deprescribing has been recognised.^9^ The World Health Organisation (WHO) has included deprescribing as a fundamental component of safe medication management, stating the process should be ‘as robust as that of prescribing’.^10^ Within certain care settings, and patient demographic groups, the process and practicalities of deprescribing has received much research attention.^11–15^ In the context of palliative care, where polypharmacy is common and medication burden is high,^16,17^ there remains limited knowledge around how to approach deprescribing decision-making.

The focus of recent deprescribing studies in this area has centred on wider issues of *identifying* polypharmacy in palliative care.^18,19^ However, little is known about the specific challenges of, and preferences towards, *decision-making* to support deprescribing for people in receipt of palliative care.^20^ While decision-making preferences have previously been considered in older people,^21^ there has not yet been work focusing solely on people with a life-limiting illness. This qualitative study aims to address this knowledge gap by exploring the perspectives of people in receipt of palliative care, alongside their family members involved in providing support with medications.

## Methods

### Design

A qualitative semi-structured interview study, involving people in receipt of palliative care and their family member(s), underpinned by reflexive thematic analysis.^22^

### Setting

Data for this study were collected from people in receipt of palliative care who resided in the North East of England – there were two hospitals and three hospice sites involved.

### Population

Inclusion criteria comprised: people in receipt of palliative care, aged 18 years and older, and their family member(s), friend(s) and/or informal carer(s) involved in supporting their care. People in receipt of palliative care were interviewed alone or with their family member(s), friend(s) and/or informal carer(s) as a dyadic interview. Eligibility criteria was based around participants being in receipt of any aspect of care from the hospital/hospice sites, including inpatient, outpatient, day therapy, community or home-based settings, and needed to be taking at least one medication at the time of interview. Participants were excluded from the study if they were receiving end-of-life care or were in the dying phase of their illness; this was assessed by each of our clinical collaborators at the study sites.

### Sampling

Purposive sampling was used to recruit participants and to ensure representation across a variety of clinical conditions that required palliative care treatment; participants were also sampled by age and sex assigned at birth to ensure maximum variation. Interviews were conducted across a number of settings (including: inpatient and outpatient wards at hospices, and participant own homes).

### Recruitment

There was no relationship established between the researchers conducting the interviews (ZB, CLR and AR-B) and participants before study commencement or recruitment. In all instances, healthcare professionals involved in the person’s usual care acted as gatekeepers in the recruitment process, by introducing the research topic to those eligible (authors JE, RQ, LB and EMcD). All interested participants were provided with an information sheet and consent form detailing the purpose of the research – those who expressed an interest and gave their informed written consent were enrolled in the study. This approach was used for all participants, with the option of involving a family member given to all.

### Data collection

In-depth semi-structured interviews were conducted by three researchers within the team (AR-B, CLR and ZB, three female researchers with expertise in qualitative methodologies); data collection was conducted between May and December 2023. Interviews took place in-person, with all participants offered the choice of a preferred location (for instance, at home or in a clinical setting) and preferred format of interview (for instance, whether to undertake it as a dyad with a family member involved, or as a one-to-one participant-only interview). The semi-structured interview topic guide (Supplemental File) was developed based on two pilot interviews and covered key issues already identified in the existing literature.^23^ The themes of questions within the topic guide remained the same across all interviews, regardless of whether the interview was conducted as a dyad or not. The topic guide was informed by the lived experiences of two patient and public research champions involved as co-authors of this study (RB and AB). The consolidated criteria for reporting qualitative research checklist (COREQ) was followed for this work (Supplemental File). All semi-structured interviews were audio-recorded to enable data analysis. The audio files were encrypted and transcribed verbatim. All interview data were anonymised at the point of transcription. Participants did not provide comment on the transcripts nor feedback on results.

### Data analysis

Following a reflexive thematic analysis approach, as defined by Braun and Clarke,^24,25^ the principle of constant comparison guided an iterative process of data collection and analysis. Reflexive thematic analysis was performed by AR-B: a close and detailed reading of the transcripts enabled familiarisation with the data; initial descriptive codes were identified in a systematic manner across the data sets; these were then sorted into common coding patterns, which enabled the development of analytic themes from the data; the themes were reviewed, refined and named once coherent and distinctive.^22^ Authors CLR and AT supported these steps, through discussion. Post-interview field notes enhanced the reflective process and enabled inductive and iterative analysis. NVivo (version 12) software was used to facilitate data management. The research team were in agreement that data sufficiency occurred after 25 semi-structured interviews; recurring similarity within participant responses, with no new concepts discussed, guided this decision. To ensure confidentiality when using direct participant quotes within this work, non-identifiable pseudonyms were used.

### Ethical approvals

This study was granted ethical approval by the UK National Health Service (NHS) Health Research Authority (reference 305394, date approved: 08.04.2022, South Birmingham REC).

## Results

### Participant characteristics

Twenty-five people in receipt of palliative care were recruited and interviewed for this study, alongside 10 of their family members who were involved in their care (Table 1: participant characteristics). There was diversity across patient age, sex and the clinical conditions for which they were in receipt of palliative care, which spanned various cancers, heart failure, motor neurone disease, pulmonary fibrosis and chronic obstructive pulmonary disease. Thirteen interviews were undertaken individually with the patient alone; 12 interviews were undertaken as dyads (i.e. where the patient and a family member were interviewed together, at the same time). The setting of each interview varied, to accommodate clinical need and patient preference, and included: hospice outpatient day units (*n* = 11), hospice inpatient wards (*n* = 4, in receipt of symptom management and/or respite care), care home (*n* = 1) and patients’ own homes (*n* = 9). Most commonly, participants took 15–20 prescribed medications per day (*n* = 11). There were no refusals to partake, participant drop outs or repeat interviews.

**Table 1.** Participant demographics.

Participant demographics	*n* = 25
Gender	
Male	10
Female	15
Age	
30–39 years	1
40–49 years	1
50–59 years	5
60–69 years	2
70–79 years	13
80–90 years	3
Self-reported ethnicity	
White British	25
Number of prescribed medications taken (per day)	
1–4	3
5–9	3
10–14	11
15–19	3
20–25	4
Unknown	1
Condition for which palliative care is required	
Cancer	13
Heart failure	5
Motor neurone disease	3
Constructive obstructive chronic disease (COPD)	1
Myeloma	1
Pulmonary fibrosis	1
Pulmonary hypertension	1
Setting/location of the interview	
Hospice outpatient unit	11
Patient home	9
Hospice inpatient ward	4
Care home	1
Family members involved in dyad interviews	(*n* = 12)
Wife	7
Daughter	2
Husband	1
Mother	1
Son	1

**Figure 1.**
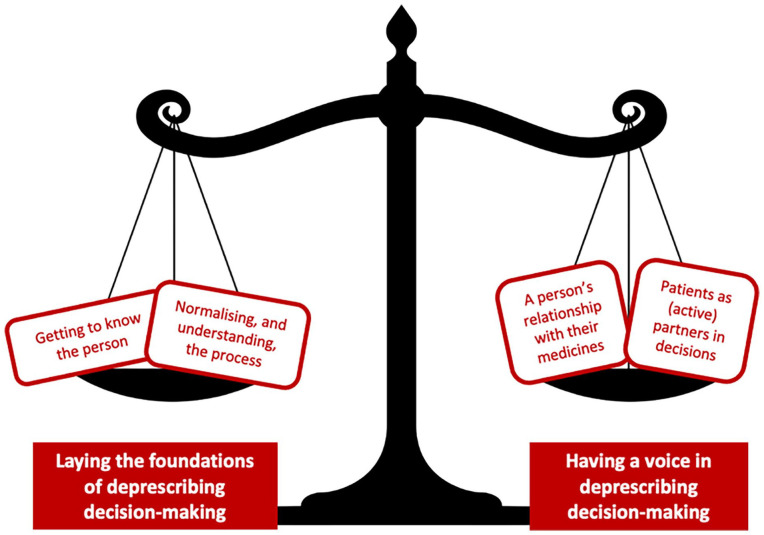
Supporting deprescribing decision-making for people in receipt of palliative care: complementing the need to lay the foundations of deprescribing decision-making alongside enabling people to have a voice in deprescribing decision-making.

Throughout the semi-structured interviews, participants described what they viewed as the ‘ideal’ approach to take when considering deprescribing decision-making; this was informed by participants sharing their perspectives, either as having had direct experience of deprescribing, or through their opinions of how this could or should be approached. There was a clear narrative which appreciated the need to complement patient understanding of the process of deprescribing, alongside actively involving patients within medicines decision-making; these two considerations appeared to underpin what was perceived as a successful, person-centred and shared approach to best support deprescribing for people in receipt of palliative care. Two overarching themes (and subsequent sub-themes) were developed which reflected and acknowledged this, centring on: (i) laying the foundations of deprescribing decision-making and (ii) having a voice in deprescribing decision-making (Figure 1).

#### Theme 1: Laying the foundations of deprescribing decision-making

##### Getting to know the person

Participants felt it important that any healthcare professional they were having a deprescribing conversation with took the time to get to know them as an individual person, ideally over a period of time, before making deprescribing recommendations. Trusting a professional’s judgement about deprescribing medicines was vital, and it appeared this trust stemmed from building a rapport with someone and ‘*feeling like (the healthcare professional) knew the background about me and then know what decisions are right for me*’ (interview 4, Brenda, person with cancer). This approach was preferred over having conversations around deprescribing with someone they were unfamiliar with. This was described by one participant as ‘*working with someone, together as a team*’ (interview 19, Paddy, person with chronic obstructive pulmonary disease) and echoed by another when describing a trusting relationship with a healthcare professional who was ‘*genuinely interested in me as a person (meant) I knew they was trying to understand the best way forward for me*’ (interview 18, Peter, a person with heart failure). Similarly, one family member remarked how important continuity in the medical team is when it comes to laying foundations of trust; this example followed a recent consultation with a locum oncology healthcare professional who ‘*had no knowledge of (patient’s) history – she didn’t know what it was all about*. . . *they should at least be aware*’ before proposing changes to medications (interview 25, family member of a person with cancer).

Participants also saw this individualised care approach as a demonstration that healthcare professionals were focused on helping their quality of life to be as best as possible through deprescribing. In essence, if patients felt ‘*like somebody has got the time for me*’ and was ‘*listening to me and genuinely trying to work out what the best combination of medications are*’ then they described feeling more assured in the decisions being made (interview 7, Eileen, person with cancer).

I did ask (the healthcare professional), I said ‘I don’t just want any doctor talking to me about this because they don’t understand what’s wrong, they don’t know’ and (the healthcare professional) said ‘No bother, you’ll only see me’ (interview 10, Betty, cancer)

### Normalising, and understanding, the process of deprescribing

Gaining a greater understanding of the process of deprescribing was deemed a priority by participants. A number of patients and their family members described feeling ‘*frightened to come off (medication) because they’ve been on it a long time*’ (interview 24, family member of a person with cancer) and ‘*I’d be frightened in case the pain comes back if I stop taking them*’ (interview 4, Brenda, person with cancer) when the concept of deprescribing was initially discussed. It was recognised, however, that the more the concept of deprescribing was discussed, the more reassured people felt. One participant perceived the approach of deprescribing to be one of flexibility – where they stated that decision-making conversations around medications does not always have to reach a finite end, instead, it could be viewed as an option to ‘*take a break from the meds, pause it and see*’ (interview 8, Maureen, person with cancer).

(Deprescribing conversations) definitely made me reflect a lot about ‘why am I taking them (medication)?’ and actually, ‘is there a moment when I could come off it, have a break, go back on?’ (interview 21, Dave, pulmonary hypertension).

This experience was not echoed widely across all participants, however. Several people in receipt of palliative care discussed how prescribers would routinely make changes to medications without fully explaining the rationale behind such decisions. One person with cancer described how ‘*they would tell me that they were going to try this new tablet – they would tell me they were going to change it but nothing else, that’s it*’ (interview 9, Joy, person with cancer). Another recognised that there was a need for healthcare professionals ‘*to explain things better*. . . *I have absolutely no problem with deprescribing at all (but) there needs to be a lot of work to help patients understand it, and so you work with (patients) rather than telling them*’ (interview 24, Helen, person with cancer). Fundamentally, acknowledging what people perceived deprescribing to involve was viewed as an essential precursor to further people’s understanding of the approach, enable shared decision-making, as well as to alleviate and/or address concerns.

#### Theme 2: Having a voice in deprescribing decision-making

##### A person’s relationship with their medicines

Considering an individual’s relationship with their medications appeared key prior to embarking on conversations around deprescribing – reasons for this centred around appreciating the hopes and expectations of particular medication(s). Many participants described only taking medication ‘*because it is given for a reason and, I would hope, that reason was to enhance whatever you’ve got*’ (interview 24, Helen, person with cancer). Others who shared the same view stated how they ‘*take them (medications) all for a reason, and each reason is important*’ (interview 23, Katherine, person with motor neurone disease). However, as soon as an individual felt that this reason was no longer beneficial, their relationship with medications appeared to change. For example, one patient described feeling ‘*very happy*’ at the prospect of deprescribing medications that were no longer clinically essential or beneficial, stating ‘*if I don’t need it, I don’t need it. I won’t miss it – it’s one less (tablet) to take*’ (interview 5, Eoin, person with cancer). There appeared a clear balance to be struck between medication being prescribed and taken, acknowledgement of the benefits to be gained from it, and the consequence on a person’s quality of life.

I think in terms of where I’m at in my life. . . the time that I’ve got left has got to be useful time, you know. (The medication) can’t just be keeping me alive, because that’s not good for me, not good for my friends. It has to be useful. So, if they tell me ‘Oh, we’ve got this great drug but it does mean that you’re going to have to stay in bed for the rest of your life’ I’d think ‘is it worth it? I’m going to decline that drug’ . . . I’m in more of a position to say I don’t want that drug (interview 6, Sarah, cancer).

Discussions about relationships with medication also extended to family members and caregivers. One person, who was an informal carer for her mother with pulmonary fibrosis, described it essential to regularly review the appropriateness of medications. What was once viewed as ‘*essential medicines, or what we thought them to be*’ at the start of treatment soon shifted after witnessing their mother be prescribed ‘*tablet after tablet*. . . *she was overmedicated. I think this is true for a lot of people, not just old people*. . . *the experts treat the problem, there’s X treatment coming from one box and rheumatoid treatment from another box*. . .’ (interview 22, daughter of a person with pulmonary fibrosis). It appeared that the balance between rationalisation of medications to avoid polypharmacy, whilst ‘*keeping their pain managed, he cannot go on being in pain or doing without that tablet*’, was a key priority (interview 3, wife of a person with cancer).

### Patients as (active) partners in decisions

Several participants described preferences to being involved in decision-making about medication. It appeared there was a balance to be struck in consultations, where the relationship between patient-professional (and family member/carer) was viewed as ‘fluctuating’ depending on each individuals’ need. Some described wanting healthcare professionals to ‘*check in and ask the question*’ about deprescribing, stating examples of ‘*saying* ‘*how are you getting on? Do you think you should be reducing this and stopping this?*” (interview 4, person with cancer). Others recognised that not everyone would not feel ready to reduce, review or stop their medications, but acknowledged that assessing a person’s readiness for change was important. One patient with cancer described a feeling of empowerment that came from deprescribing their medications, stating ‘*it means that (patients) can start questioning things more*. . . *feeling part of that decision-making process*. . . *so I feel I’ve made that decision with them*’ (interview 7, Eileen, person with cancer).

Many participants and family members viewed themselves as ‘*partners in decisions*’ and wished to have their own voices heard as ‘*part of the team*’, alongside their healthcare professionals (interview 10, relative of a person with cancer). Others described initial preferences to solely ‘*trust the clinical expertise of the ones who make the decisions*’; this was particularly prominent for some patients who described their awareness of ‘*the (medical professionals) as the experts*. . . *I just take what I’m told*. . . *it would be difficult for me to make constructive decisions to whether to take them or not*’ (interview 21, Dave, person with pulmonary hypertension). Over time, however, there appeared a shift in this dynamic towards a less paternalistic approach to decision-making – instead, people began to describe their wishes to speak up as an active partner in decisions. Alongside their husband, one patient with cancer explained this from a perspective of trying to retain some form of control. They both echoed that ‘*the element of having control, having something where you can still have your voice in*. . . *certainly have the dialogue so you’re part of it (making decisions)*. . .*that’s important*’ (interview 24, Helen, person with lung cancer and their husband). This aspect of inclusion was deemed a significant ‘*step forward*’ towards person-centredness during a time where, typically, there is little that patients can control or influence (interview 6, Sarah, person with cancer).

Participants shared examples of times when they or members of their family proactively initiated conversations around deprescribing by prompting ‘*do I actually even need all of these tablets?*’ (interview 21, Dave, person with pulmonary hypertension). Family members described advocating for their relatives who were in receipt of palliative care, with many empathising with the burden of medication that a person was taking each day. Efforts to understand medicine indications helped to question ‘*whether they were essential for him (husband) to keep taking*’ (interview 15, wife of a person with heart failure).

The active inclusion, just the way that they were having a role shift from them standing at the end of the bed, to them making decisions with you. They were just explaining it and explaining the reason (medication rationale), you know, it’s just a simple. . . they probably don’t care what I think really, but, just by saying it, it makes you feel included (interview 6, Sarah, person with cancer)

## Discussion

### Main findings

This study provides a unique stance on the existing evidence base around deprescribing decision-making, by focusing specifically on the context of deprescribing medications for people in receipt of palliative care. The voices of those with lived-experience of palliative care, alongside their family members, feature at the centre of this work which has enabled a greater understanding of deprescribing decision-making approaches most suitable for this group of people. Across all interviews, there was a consistent emphasis placed on the need for healthcare professionals to approach deprescribing decision-making in a way that complemented: (i) laying the ‘optimal’ foundations conducive to successful deprescribing discussions, alongside (ii) supporting the person in receipt of palliative care (and their family members, if appropriate) to have an active voice in decisions being made.

### What this study adds

Person-centred, individualised care approaches have been researched within wider healthcare literature,^26^ and most recently in broader deprescribing approaches.^27,28^ Echoing findings across other cohorts, such as the ageing population^29^ and those with chronic illness,^30^ continuity and familiarity of a trusted healthcare professional was considered vital when making decisions about deprescribing in palliative care.^31^ The emphasis of placing the person at the centre of decision-making, and fostering a culture of *shared* decision-making, appeared to be enabled by prescriber familiarity.^32^ Amongst this cohort of people in receipt of palliative care, it was deemed essential to establish each individuals’ relationship with their medication within deprescribing decision-making consultations.^21^ Similar to previous research that approached deprescribing using shared decision-making for older adults,^33^ one recommendation arising from this work is that shared decision-making can facilitate people in receipt of palliative care to find their voice and play an (active) role within deprescribing.

Similar to deprescribing in chronic health conditions, there was clear interplay between patient fears of deprescribing versus their understanding of the process.^34,35^ Previous evidence has demonstrated that the more a person understands about the rationale for stopping a medication, the more likely they are to accept deprescribing decisions.^36^ The terminology and language used when (de)prescribing medications warrants further investigation; future studies should seek to identify and recommend terminology that supports a wider understanding of ‘indefinite’ prescribing, whilst alleviating any fears or concerns that may accompany stopping a medicine. The use of such terminology may better manage expectations and improve the acceptability of deprescribing decisions in light of clinical disease trajectory – for the patient, as well as family members.

The involvement of family members within deprescribing discussions in this study echoed previous findings, where ‘successful’ deprescribing interventions for older people noted the inclusion of family as key.^36^ Family members have described adopting advocacy roles as carers, often initiating and prompting conversations about medication appropriateness.^37^ In wider studies, family members have had varying degrees of involvement, from being equal partners in supporting their patient companions, to being fully responsible and active, independent carers.^21,38,39^ The connection between deprescribing decision-making from a family or carer perspective could be further explored; in particular, how best to frame and approach those conversations to enable informed and shared decision-making about deprescribing to take place.^34,40–42^ Through use of co-design methodologies, future research could further explore the interplay of deprescribing decisions with all relevant stakeholders.

Future studies could also seek to explore deprescribing decision-making in relation to a person’s readiness for change while receiving palliative care. Findings from this study illustrated changing perspectives that a patient, and their family members, may have towards deprescribing at varying time points within their disease trajectory, towards the end-of-life. Whilst there have been recent efforts undertaken to explore generalised links between readiness for change, behavioural change theory and deprescribing interventions,^43–45^ there remains a distinct gap in knowledge specific to palliative care – both in terms of perspectives from people involved in receiving, and those providing, palliative care. The trajectory of end-of-life care is unique to each person, giving rise to the requirement to better understand how person-centred decision-making efforts can be tailored to proactively deprescribe medicines.

### Strengths and limitations of the study

The study population included people with different life-limiting illnesses and was inclusive of people in receipt of palliative care, alongside their family members, to appreciate breadth of views and experiences. The semi-structured interviews captured perspectives of people receiving palliative care in inpatient wards and outpatient clinics, or in their own homes. Despite the sample being reflective and characteristic of the demographics of people typically in receipt of palliative care within the region,^46^ the research team acknowledge that the inclusion of people from ethnic minority communities would bring valuable insights when striving to deliver deprescribing practices that are person-centred, inclusive and culturally competent. A recent scoping review highlighted the paucity of deprescribing research being conducted in ethnically diverse populations outside of the United States, as well as limited focus on deprescribing decision-making involving people with non-Christian religious beliefs.^47^ Consideration could also be given, in future research, to explore deprescribing decision-making with participants aged outside of the age-range of this study; for example, factors such as health literacy, independence, perceptions of medications and the impact on lifestyle could affect perspectives on deprescribing across different age groups.^48–50^ Tailoring discussions to address these differences could support enhanced patient engagement and alignment of decisions with individual values.

## Conclusion

Despite the significant polypharmacy burden and rationale to reduce or stop medications for people in receipt of palliative care, deprescribing decision-making in this context remains an under researched topic. These findings demonstrate the need to complement patient understanding of the process of deprescribing, alongside actively involving patients within medicines decision-making; however, there still remains a gap in knowledge about how best to practically and effectively approach this. Future research should seek to further explore approaches of such decision-making processes, considering the views of all parties involved including people receiving palliative care, their family members or caregivers, alongside healthcare professionals. Co-design strategies could offer one approach towards better understanding and delivering on deprescribing decision-making within palliative care contexts. Additionally, the perspectives of people from minoritised communities could provide valuable insight, and offer recommendations, on underpinning such approaches with cultural competence.

## Supplemental Material

sj-docx-1-pmj-10.1177_02692163251327900 – Supplemental material for ‘Do I actually even need all these tablets?’ A qualitative study exploring deprescribing decision-making for people in receipt of palliative care and their family members Supplemental material, sj-docx-1-pmj-10.1177_02692163251327900 for ‘Do I actually even need all these tablets?’ A qualitative study exploring deprescribing decision-making for people in receipt of palliative care and their family members by Anna Robinson-Barella, Charlotte Lucy Richardson, Zana Bayley, Andy Husband, Rona Bojke, Andy Bojke, Rachel Quibell, Lisa Baker, Emma McDougall, Catherine Exley, Barbara Hanratty, Joanna Elverson, Jesse Jansen and Adam Todd in Palliative Medicine
