# Supplementary material for: ‘Do I actually even need all these tablets?’ A qualitative study exploring deprescribing decision-making for people in receipt of palliative care and their family members
Source: Palliat Med. 2025 Apr 1;39(5):543–52. doi: 10.1177/02692163251327900 (PMC12033382; doi:10.1177/02692163251327900)
Supplement: sj-docx-1-pmj-10.1177_02692163251327900 – Supplemental material for ‘Do I actually even need all these tablets?’ A qualitative study exploring deprescribing decision-making for people in receipt of palliative care and their family members [file sj-docx-1-pmj-10.1177_02692163251327900.docx]

**Item 1:**

**COnsolidated criteria for REporting Qualitative studies (COREQ): 32-item checklist.**

| **Number** | **Item** | **Guide questions / description** | **Reported on manuscript page** |
| --- | --- | --- | --- |
| **Domain 1: research team and reflexivity** | | | |
| **Personal characteristics** | | | |
| 1 | Interviewer | Which author(s) conducted the interviews? | 5-6 |
| 2 | Credentials | What were the researcher’s credentials? *E.g., PhD, MD* | 5-6 |
| 3 | Occupation | What was their occupation at the time of the study? | 5-6 |
| 4 | Gender | Was the researcher male or female? | 5-6 |
| 5 | Experience and training | What experience or training did the researcher have? | 5-6 |
| **Relationship with participants** | | | |
| 6 | Relationship established | Was a relationship established prior to study commencement? | 5 |
| 7 | Participant knowledge of interviewer | What did the participants know about the researcher?  *E.g., reason for doing the research* | 5 |
| 8 | Interviewer characteristics | What characteristics were reported about the interviewer?  *E.g., bias, assumptions, reasons and interests in the research topic* | 5 |
| **Domain 2: study design** | | | |
| **Theoretical framework** | | | |
| 9 | Methodological orientation and theory | What methodological orientation was stated to underpin the study?  *E.g., grounded theory, ethnography, discourse analysis* | N/A |
| **Participant selection** | | | |
| 10 | Sampling | How were participants selected? *E.g., purposive, convenience, consecutive* | 5 |
| 11 | Method of approach | How were participants approached? *E.g., face-to-face, telephone, email* | 5 |
| 12 | Sample size | How many participants were in the study? | 6-7, Table 1 |
| 13 | Non-participation | How many people refused to participate or dropped out (with reasons)? | 7 |
| **Setting** | | | |
| 14 | Setting of data collection | How was the data collected? *E.g., home, clinic, workplace* | 5-6, Table 1 |
| 15 | Presence of non-participants | Was anyone else present besides the participant and researcher? | N/A |
| 16 | Description of sample | What are the important characteristics of the sample? *E.g., demographic data* | 6-7, Table 1 |
| **Data collection** | | | |
| 17 | Interview guide | Were questions and prompts provided by the authors? | Item 2, Supplementary file |
| 18 | Repeat interviews | Were repeat interviews carried out? If yes, how many? | N/A |
| 19 | Audio/visual recording | Did the researcher use audio or visual recording to collect the data? | 5-6 |
| 20 | Field notes | Were field notes made during/after the interview? | 5-6 |
| 21 | Duration | What was the duration of the interviews? | Item 3, Supplementary file |
| 22 | Data saturation | Was data saturation discussed? | 6 |
| 23 | Transcripts returned | Were transcripts returned to participants for comment/correction? | 6 |
| **Domain 3: analysis and findings** | | | |
| **Data analysis** | | | |
| 24 | Number of data coders | How many data coders coded the data? | 6 |
| 25 | Description of the coding tree | Did authors provide a description of the coding tree? | N/A |
| 26 | Derivation of themes | Were themes identified in advance or derived from the data? | 6 |
| 27 | Software | What software, if applicable, was used to manage the data? | 6 |
| 28 | Participant checking | Did participants provide feedback on the findings? | 6 |
| **Reporting** | | | |
| 29 | Quotations presented | Were participant quotations presented to illustrate the themes / findings? Was each quotation identified? E*.g., participant number* | 12-16 |
| 30 | Data and findings consistent | Was there consistency between the data presented and the findings? | 16-18 |
| 31 | Clarity of major themes | Were major themes clearly presented in the findings? | 12-16, Figure 1 |
| 32 | Clarity of minor themes | Is there a description of diverse cases or discussion of minor themes? | 12-16, Figure 1 |

**Item 2:**

**Semi-structured interview topic guide**

The semi-structured interview questions were based around the following topic areas:

1. Can you tell me a little about yourself and why you have decided to take part in this study?

2. Talk me through your experiences of using your medicines

*Prompts:* How many do you use? Do you know what they are for? How do you fit taking medicines around your daily routine?

3. Talk me through the reasons why you take your medicines – do you value certain medications over others?

*Prompts:* consider goals, values and preferences; reasons for taking medications; how are medications prioritised?

4. Have you ever experienced side effects from your medicines? If so, what were they?

*Prompts:* risks versus benefits of treatment; management of side effects; impact on daily living/medication taking behaviour

5. Is there anything you would like to change about your medication (if this was possible?)

*Prompts:* consider burden from medication, possible reduction or deprescribing, more medication for acute symptom management?

6. When deciding if you should take a medicine (or not), what sort of things help you decide if you should take it?

*Prompts:* benefit versus harm? Use of evidence? Family involvement? Advice from healthcare professional?

7. From your previous experience of deciding to take a medicine, how involved were you in making a decision?

*Prompts:* importance, explored shared-decision making, differ according to medicines? What could be done to help make a decision? Involvement of other stakeholders.

8. Is there anything else you think is important that you would like to share with us?

**Item 3:**

**Further interview data**

| Participant number | Interview duration (minutes) |
| --- | --- |
| 1 | 34 |
| 2 | 25 |
| 3 | 39 |
| 4 | 37 |
| 5 | 11 |
| 6 | 37 |
| 7 | 41 |
| 8 | 29 |
| 9 | 37 |
| 10 | 51 |
| 11 | 32 |
| 12 | 13 |
| 13 | 51 |
| 14 | 30 |
| 15 | 30 |
| 16 | 45 |
| 17 | 45 |
| 18 | 45 |
| 19 | 37 |
| 20 | 33 |
| 21 | 48 |
| 22 | 50 |
| 23 | 21 |
| 24 | 43 |
| 25 | 44 |
